# Clinical performance of tooth implant–supported removable partial dentures: a systematic review and meta-analysis

**DOI:** 10.1007/s00784-022-04622-7

**Published:** 2022-07-15

**Authors:** Pedro Molinero-Mourelle, Frank Bischof, Burak Yilmaz, Martin Schimmel, Samir Abou-Ayash

**Affiliations:** 1 Department of Reconstructive Dentistry and Gerodontology, School of Dental Medicine, University of Bern, Freiburgstrasse 7, Bern, 3010 Switzerland; 2 Department of Restorative, Preventive and Pediatric Dentistry, School of Dental Medicine, University of Bern, Bern, Switzerland; 3 Division of Restorative and Prosthetic Dentistry, The Ohio State University College of Dentistry, Columbus, OH USA; 4 Division of Gerodontology and Removable Prosthodontics, University Clinics of Dental Medicine, University of Geneva, Geneva, Switzerland

**Keywords:** Partially edentulous, Partial denture, Dental implant, Implant abutment design, Overdenture

## Abstract

**Objective:**

To assess the clinical performance of tooth implant–supported removable partial dentures in terms of abutment survival in relation to the attachment system used.

**Methods:**

An electronic search in MEDLINE/PubMed Web of Science and Cochrane Central Register of Controlled Trials databases was performed. The methodological quality of the studies was assessed using the Newcastle–Ottawa Scale. Survival rates after 3 years and 5 years, loss, and complication rates per 100 years were estimated by Poisson regression.

**Results:**

A total of twelve studies were included; eleven studies were used for the meta-analysis. Survival analysis for mixed attachments showed an estimated survival rate of 100% after 3 years and 5 years. For uniform attachments, the estimated survival rate was 99.3% after 3 years and 98.8% after 5 years. Tooth abutment survival analysis for mixed attachments estimated a survival rate of 95% after 3 years and 91.7% after 5 years: Uniform attachments reached a survival rate of 97.2% after 3 years and 95.4% after 5 years. The prosthetic survival rate was 100% for mixed and uniform abutments after 3 years and 5 years of function.

**Conclusions:**

Tooth implant–supported removable partial dentures can be considered as a reliable option with excellent prosthetic and implant survival rates and favorable rates for the abutments after 3-year and 5-year follow-ups. Complications may be reduced when 5 or more abutments are used.

**Clinical relevance:**

Tooth implant–supported removable partial dentures are a favorable and potential alternative to restore a partially edentulous arch by optimizing the number and distribution of abutments.

**Supplementary Information:**

The online version contains supplementary material available at 10.1007/s00784-022-04622-7.

## Introduction

In past decades, an improvement in dental maintenance occurred in industrialized countries, resulting in a decreased incidence of tooth loss [1]. Nevertheless, there is an increasing demand for prosthetic rehabilitation of patients 65 years or older with ≥ 7 missing teeth [1]. Available treatment options depend on the number, prognosis, and location of remaining teeth; patients’ demands; expectations; and financial possibilities [2–4]. In this respect, the Eurostat data shows a correlation between economic recession and unmet medical and/or dental visits due to financial reasons [5]. Conventional and implant-supported fixed partial dentures (FPDs) are accessible mainly to patients from middle and higher-income groups. Consequently, more affordable treatment options are needed to address the needs of patients from lower-income groups. In situations with multiple missing teeth and/or extended soft tissue defects, rehabilitation with removable partial dentures (RPDs) may be a cost-effective alternative to FPDs. However, RPDs, especially with distal extensions, are prone to adjustments such as relining or fracture repairs [6]. Depending on the number and distribution of remaining teeth, combination of implant and teeth has been proposed for implant-assisted removable partial dentures (IARPDs) [7, 8]. With IARPDs, two types of implant abutments are commonly used: individualized (i.e., double crowns) or prefabricated (i.e., ball/stud) abutments. These abutment options are combined with individual tooth abutments, including double crowns, clasps, or root copings [1, 9, 10]. This “intermediate” treatment alternative offers the possibility to restore a partially edentulous arch by optimizing the number and distribution of abutments [11]. Using implants in strategic positions improves patient satisfaction and masticatory performance [11, 12]. Furthermore, a removable option facilitates the compensation for soft tissue loss [13], results in lower cost per replaced tooth compared to FDPs, and is adjustable, in case further tooth loss occurs [14]. Nevertheless, this option requires teeth, with good periodontal prognosis to provide favorable distribution of occlusal forces, and sufficient bone that allow the implant placement in prosthetically and biomechanically favorable positions [2, 15].

The clinical performance of implant-supported FPDs and implant-retained overdentures has been extensively studied [16–18]. However, there is limited evidence on the clinical performance of IARPDs, specifically, regarding the attachment system selection in tooth implant combination situations. Hence, the aim of this systematic review and meta-analysis was to assess the current evidence on clinical performance of IARPDs in terms of teeth and implant abutment survival when uniform or mixed attachment systems are used. In particular, the influence of identical attachment type on teeth and implants (uniform attachment) was aimed to be compared with that of different attachments (mixed attachments) on teeth and implants. In addition, implant and tooth abutment survival rates and success, prosthetic survival rates, and complications were aimed to be analysed.

## Material and methods

### Registration

The present systematic review was registered and allocated the identification CRD42020176146 in the International Prospective Register of Systematic Reviews (PROSPERO) hosted by the Centre for Reviews and Dissemination, University of York, National Institute for Health Research (UK).

### Protocol development and Population, Intervention, Comparison, Outcome question

This systematic review was conducted according to the Cochrane guidelines and in accordance with all Preferred Reporting Items for Systematic Reviews and Meta-Analyses (PRISMA) [19]. It was designed according to the Population, Intervention, Comparison, Outcome (PICO) model:

- *Population*: partially edentulous maxilla and/or mandible with an IARPD in at least one arch
- *Intervention or exposure*: uniform implant and tooth attachment systems
- *Comparison*: mixed attachment systems
- *Outcome*: survival rates of implant and tooth abutments, prosthetic survival rates and complications

The resulting PICO question was: Is there a difference in abutment survival when uniform attachments are used on teeth and implants compared with mixed attachments in partially edentulous patients rehabilitated by using IARPDs?

### Synthesis of the results

For this systematic review, abutment/prosthetic survival was defined when teeth, implants, and/or prostheses were functional and in situ including intraoral or extraoral, direct or indirect maintenance and repairs without the need of a new prosthesis fabrication. The term “uniform attachments” was used to define those prostheses that had the same attachment system on both abutments (e.g., ball abutments on teeth and implants), and the term “mixed attachment” was used for those with different attachment systems on different abutments in the same prosthesis (e.g., ball abutments on implants and telescopic crowns on teeth).

The biological and technical/mechanical complications were identified according to the following situations:

### Biological complications

- Minimal: peri-implant mucositis, gingivitis, or sore spots
- Moderate: peri-implantitis, periodontitis, caries, or endodontic pathology
- Severe: tooth root/crown fracture, endodontic treatment failure, or tooth/implant loss

### Technical/mechanical complications

- Minimal: screw loosening, relining, sore spot adjustment, loss of retention, matrix activation, or change and occlusal attachment
- Moderate: chipping/fracture of veneering material, primary abutment decementation, replacement of a damaged artificial tooth, or lost/broken retention element
- Severe: post facture, framework/base fracture, or necessity for prosthesis remake

For quantitative analysis of complications, only the reports that provided clear data on clinical parameters and follow-up for implant and/or tooth abutment and prosthesis individual analysis were included. In addition, all complications were descriptively evaluated.

### Eligibility criteria

#### Inclusion criteria

- Clinical studies of partially edentulous patients rehabilitated with IARDPs
- Randomized and controlled clinical trials, cross-sectional studies, cohort studies, case–control studies, and case series including at least 10 participants were considered.
- Clinical performance clearly documented in the study, including information on at least abutment survival
- Minimum follow-up period of 1 year
- Publications written in English, German, or Spanish

### Exclusion criteria

- In vitro or animal studies
- Patients rehabilitated with conventional RPDs
- Completely dentate and edentulous patients
- Insufficient documentation on abutment survival

### Search strategy and sources of information

Studies were identified by entering the following search terms: (partially edentulous or edentulous or jaw or partially edentulous or partial edentulism or edentulous) and (dental implants or dental prosthesis or implant-supported denture or removable partial denture or denture) and (abutments or prosthesis design or implant-abutment design) and (survival rate or complications or complications or survival or loss) (Table 1).

**Table 1:** Search strategy

Focused question (PICO)	In tooth implant–retained removable dentures, is there a difference in abutment survival using uniform attachments compared to mixed attachments on teeth and implants?	
Search strategy	#1Population	“Partially edentulous” OR edentulous OR jaw OR “partial edentulism” OR edentulous [MeSH]
	#2 Intervention or exposure	“Dental prosthesis, implant-supported” [MeSH] OR “denture, partial, removable” [MeSH] OR “removable denture” OR “removable partial denture”
	#3 Comparison	“Dental abutments” [MeSH] OR “dental clasps” [MeSH] “dental prosthesis design” [MeSH] OR “dental implant-abutment design” [MeSH] OR “dental implants” [MeSH] OR “implant-supported” OR “tooth-supported” OR “implant-retained” OR “tooth-retained” OR “tooth-implant” OR “implant-tooth”
	#4 Outcome	“Survival Rate” [MeSH] OR “Biological complications” OR “Technical complications,” “Survival,” OR “Loss”
	Search combination	#1 AND #2 AND #3 AND #4
Database search	PubMed, Web of Science, and Cochrane Library	(Partially edentulous or edentulous or jaw or partially edentulous or partial edentulism or edentulous) and (dental implants or dental prosthesis, implant-supported or denture, partial, removable or removable partial denture or denture, removable partial or dentures, removable partial or partial denture, removable or partial dentures, removable or removable partial dentures) and (dental abutments or dental class dental prosthesis design or dental implant-abutment design) and (survival rate or biological complications or technical complications survival or loss)

An electronic search was performed up to January 2022 in 3 databases: National Library of Medicine (MEDLINE [PubMed]) via Ovid, Web of Science (WOS), and Cochrane Central Register of Controlled Trials (CENTRAL). No time or language restrictions were applied. Additionally, hand-search was performed screening the publication lists of the following journals up to January 2022: *Clinical Oral Implants Research*, *Clinical Implant Dentistry and Related Research*, *International Journal of Oral & Maxillofacial Implants*, *Journal of Prosthetic Dentistry*, *Journal of Prosthodontics*, *Journal of Prosthodontic Research*, *International Journal of Prosthodontics*, *Journal of Dentistry*, *Clinical Oral Investigations*, and *Journal of Oral Rehabilitation and Gerodontology*. Moreover, references of included studies were screened to identify further potential articles. Grey literature was not searched.

### Study selection

Publications of IARPDs were included after the electronic search in databases without using any filters, and the references were assessed by using a reference manager software program (EndNote, Thomson Reuters) and identifying and eliminating duplicates. Two reviewers (P.M-M. and F.B.) independently performed the title and abstract screening. Studies that were identified as unclear were included for full-text screening. Any disagreement between the reviewers regarding article suitability was resolved by discussion, and if necessary, the senior author (S.A-A.) was consulted. When the information provided by a potentially eligible article was not clear, the authors were contacted by email. When multiple studies reported the same patient cohort, the study with longer follow-up was included. For studies reporting multiple relevant cohorts, clinical data from each group were recorded separately. The level of agreement between the reviewers for study selection process was estimated by using Cohen’s kappa statistics (k-score).

### Data collection and items

Data collection was independently performed by using a standardized electronic sheet (Excel, Microsoft), and the study data extraction included the following parameters: authors, year of publication, study design, number of patients, mean age, mean follow-up, number of implants, number of abutment teeth, implant brand, type of abutment, abutment material abutment survival, type of retention, planned number of patients, failure of abutments, and abutment/implant complications.

### Risk of bias in individual studies

The methodological quality of the selected studies was evaluated using the Newcastle–Ottawa Scale (NOS) by 2 independent reviewers (P.M-M. and F.B.). This scale includes 3 main categories: selection of study groups, comparability, and outcome. Each individual study received a maximum of 9 points [20]. In case of no consensus during the evaluation process, discrepancies were discussed with a third evaluator (S.A-A.).

### Statistical analysis

Survival rates after 3 years and 5 years, loss, and complication rates per 100 years were estimated by Poisson regression: Assuming that the total number of events follows a Poisson distribution, a regression model was used to model the rate of random events that occur in the exposure time, e.g., the complication rate over a fixed period of time. Furthermore, survival rates with 95% confidence intervals after 3 years and 5 years were calculated using the relationship between event rate and survival function (*S*) (*S*(*T*) = exp(− *T* × event rate). In addition, Poisson regression was used to estimate the rate at which events occurred (incidence rate) within subgroups and to compare the incidence rates of subgroups by calculating incidence rate ratios (IRRs) with 95% confidence intervals. All statistical tests were two-sided (*α* = 0.05). Stata/IC 16.0 for Windows (StataCorp LLC, 4905 Lakeway Drive, College Station, TX 77,845, USA) was used for statistical analysis.

## Results

### Search results

**Fig. 1:**
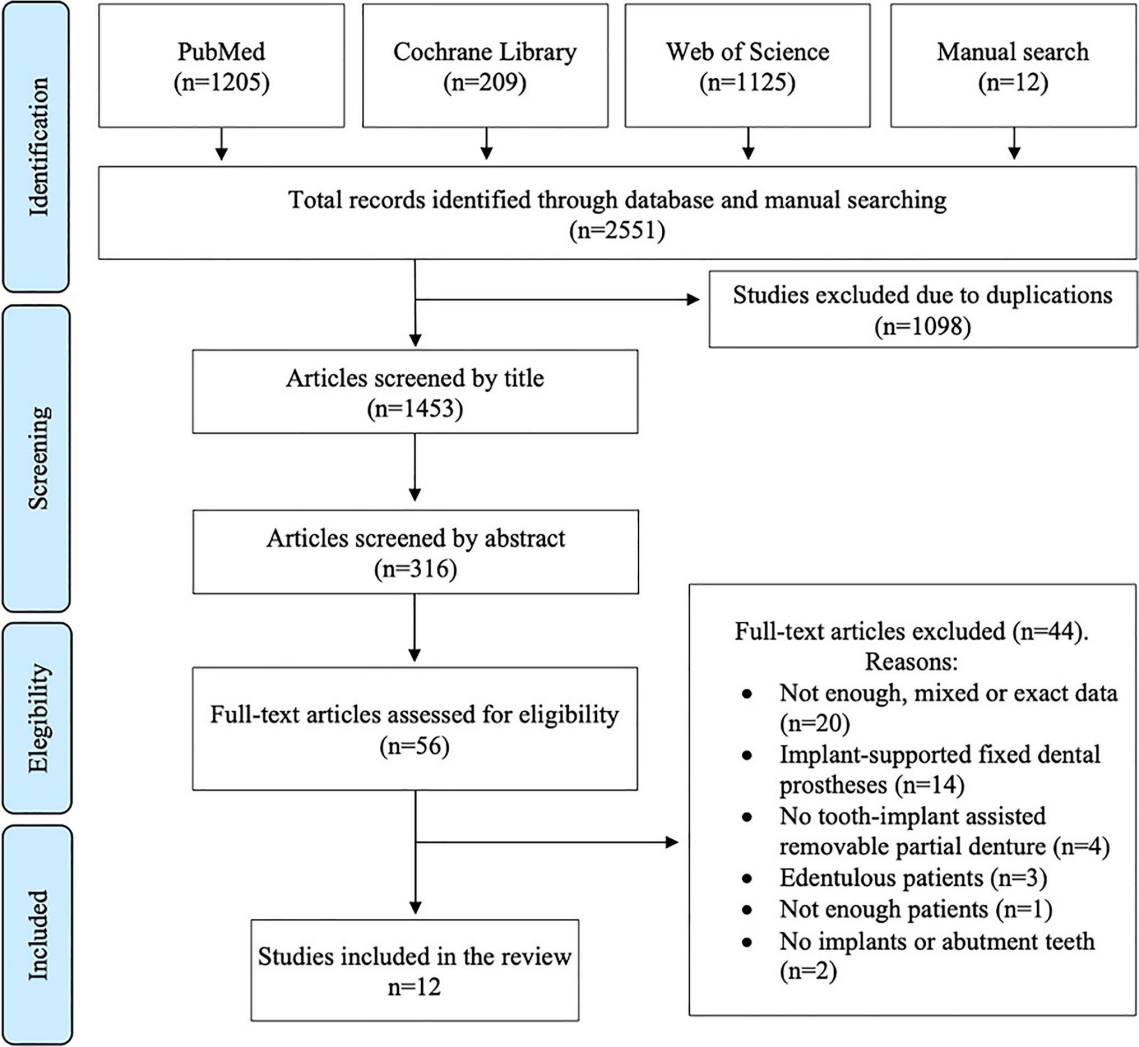
Flow chart

The initial electronic database search resulted in 2539 titles, and the manual search yielded 12 additional articles, resulting in a total of 2551 potential references. Of those, 1098 were duplicates and were removed, and finally, 1453 were screened by title (Fig. 1). The initial elimination of articles not relevant to the focus question was followed by the stepwise title and abstract screening, and a total of 56 articles were selected for full-text analysis (Annex 1). In the inter-reviewer’s agreement level, Cohen’s kappa statistic was 0.78 (substantial agreement) for title selection, 0.38 for abstract selection (fair agreement), and 0.29 (fair agreement) for full-text assessment. Whenever 2 reviewers could not find agreement on the inclusion of a study, a third independent reviewer was consulted.

Twelve studies (3 prospective and 9 retrospective clinical studies) were included with a follow-up that ranged from 24 to 180 months (Table 2). These studies reported on 408 patients (47.5% female and 52.5% male, age range of 46.8 to 71.6 years) with 359 prostheses, 902 implants, and 983 abutment teeth. Ten studies reported on telescopic crown systems [15, 21–24, 26, 27, 29, 30], and two studies reported a combination of telescopic crown and ball attachment system [25, 28] (Table 3). The authors of three studies were contacted, asking for further information in terms of prosthetic survival [9, 30] and complications [27]. Two authors replied, but were not able to provide required information, and one author did not reply.

**Table 2:** Characteristics of the included studies

Author (year)	Country	Type of study	Total patients	Patients included	Age	Sex	Follow-up (months)	Number of arches	Antagonist arch
Bernhart et al. (2012) [21]	Germany	Retrospective	63	16	63.3 ± 8.8 (41–84)	19 female, 44 male	24	16	NM
Fobbe et al. (2019) [22]	Germany	Retrospective	126	86	65.6 ± 9.1	36 female, 50 male	50.4 (6–134.4)	86	NM
Frisch et al. (2015) [23]	Germany	Retrospective	26	23	71.6 ± 8 (52.3–86.4)	15 female, 8 male	73.2 ± 45.6	23	RPD or FPD
Guarnieri and Ippoliti (2018) [24]	Italy	Retrospective	18	18	46.8 ± 6.3 (32–64)	7 female, 11 male	180	36	RPD
Hug et al. (2006) [25]	Switzerland	Prospective	46	14	67.5	7 female, 7 male	24	18	NM
Joda (2013) [26]	Switzerland	Retrospective	10	10	66.6 ± 8.6 (52–80)	5 female, 5 male	26.3 ± 7.5 (18–40)	10	NM
Kern et al. (2019) [27]	Germany	Prospective	31	29	56.7 ± 8.5	17 female, 14 male	135.6 ± 13.2 (105.6–156)	29	NM
Krennmaier et al. (2007) [15]	Austria	Retrospective	22	22	63.7 ± 7.9	14 female, 8 male	38 ± 14.6 (12–108)	22	RPD or FPD
Marotti et al. (2015) [28]	Germany	Prospective	22	11	70.4 (57–78)	6 female, 5 male	78 ± 10.8 (60–98.4)	11	CD, RPD, or FDP
Rammelsberg et al. (2014) [9]	Germany	Retrospective	61		65.4	22 female, 39 male	32.4	39	NM
Rinke et al. (2015) [29]	Germany	Retrospective	18	14	66.05 ± 8.01 or 9.01 (50.7–80.1)	11 female, 3 male	70.1 ± 36 (65.4)	14	CD, RPD, or FDP
Romanos et al. (2012) [30]	USA	Retrospective	55	55	63.51 ± 9.95 (40–84)	35 female, 20 male	61.5 (24–125)	55	NM

*NM* not mentioned, *RPD* removable partial denture, *FPD* fixed partial denture, *CD* complete denture

**Table 3:** Implants, implant abutment, and tooth abutment data of the included studies

Author and year	Number of implants	Implant manufacturer	Implant abutments system	Implant abutments survival	Implant abutments success	Number of teeth	Tooth abutments system	Tooth abutments survival	Prosthetic survival
Bernhart et al. (2012) [21]	40	Straumann and Neodent	Telescopic crowns	40 (100%)	NM	44	Telescopic crowns	44 (100%)	16 (100%)
Fobbe et al. (2019) [22]	199	Straumann	Telescopic crowns	198 (99.49%)	NM	239	Telescopic crowns	230 (96.23%)	NM
Frisch et al. (2015) [23]	61	Ankylos	Telescopic crowns	60 (98.36%)	60 (98.36%)	66	Telescopic crowns	57 (86.36%)	23 (100%)
Guarnieri and Ippoliti (2018) [24]	164	BioLok, P1H	Telescopic crowns	158 (96.34%)	NM	233	Telescopic crowns	214 (91.84%)	36 (100%)
Hug et al. (2006) [25]	20	Straumann	Ball attachment (15); telescopic crowns (5)	15 (75%)	NM	32	Root cap	31 (96.87%)	18 (100%)
Joda (2013) [26]	28	Straumann, Nobel Biocare, Ankylos	Telescopic crowns	28 (100%)	NM	28	Telescopic crowns	28 (87.5%)	10 (100%)
Kern et al. (2019) [27]	69	Straumann	Telescopic crowns	67 (97.1%)	NM	66	Telescopic crowns	56 (98.48)	29 (100%)
Krennmaier et al. (2007) [15]	60	Camlog, Genesio	Telescopic crowns	60 (100%)	NM	48	Telescopic crowns	48 (100%)	22 (100%)
Marotti et al. (2015) [28]	34	Camlog	Ball attachment, telescopic crowns	34 (100%)	NM	18	Telescopic crowns	16 (88.88%)	11 (100%)
Rammelsberg et al. (2014) [9]	93	Straumann	Telescopic crowns	NM	91 (97.84%)	107	Telescopic crowns	103 (96.26%)	NM
Rinke et al. (2015) [29]	24	Ankylos	Telescopic crowns	24 (100%)	23 (95.83)	27	Telescopic crowns	23 (85.18%)	14 (100%)
Romanos et al. (2012) [30]	110	Ankylos	Telescopic crowns	107 (97.27%)	101 (91.81%)	75	Telescopic crowns	66 (88%)	NM

*NM* not mentioned

### Quality assessment

The results from the quality assessment of included studies based on the Newcastle–Ottawa Scale for cohort studies reported six stars in two studies [25, 28], five stars in eight studies [9, 15, 21, 22, 24, 26, 27, 29], and four stars in two studies [23, 30]. These scores pointed to a medium quality of evidence among the studies reviewed (Table 4).

**Table 4:** Quality assessment of included studies using the Newcastle–Ottawa scale

Study	Selection				Comparability		Outcome			Number of stars (out of 9)
	S1	S2	S3	S4	C1	C2	E1	E2	E3	
Bernhart et al. (2012) [21]	★	0	★	★	0	0	0	★	★	5
Fobbe et al. (2019) [22]	★	0	★	★	0	0	0	★	★	5
Frisch et al. (2015) [23]	★	0	★	0	0	0	0	★	★	4
Guarnieri and Ippoliti (2018) [24]	★	0	★	★	0	0	0	★	★	5
Hug et al. (2006) [25]	★	★	★	★	★	0	0	★	0	6
Joda (2013) [26]	★	0	★	★	0	0	0	★	★	5
Kern et al. (2019) [27]	★	0	★	★	0	0	0	★	★	5
Krennmaier et al. (2007) [15]	★	0	★	★	0	0	0	★	★	5
Marotti et al. (2015) [28]	★	0	★	★	0	0	★	★	★	6
Rammelsberg et al. (2014) [9]	★	0	★	★	0	0	0	★	★	5
Rinke et al. (2015) [29]	★	0	★	★	0	0	0	★	★	5
Romanos et al. (2012) [30]	★	0	★	★	0	0	0	★	★	4

### Implant abutment survival

**Fig. 2:**
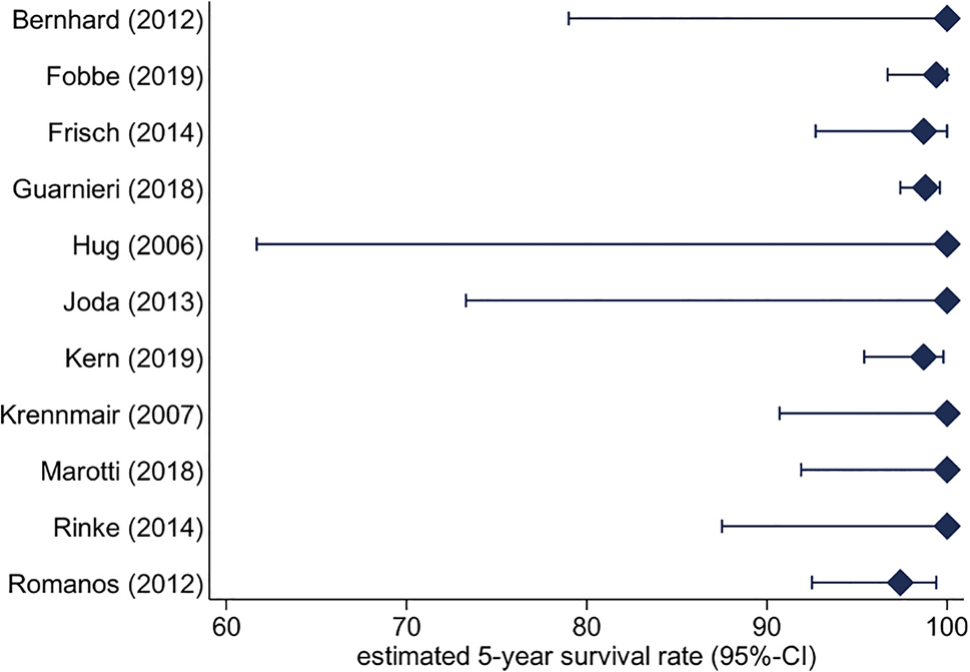
Implant abutment estimated 5-year survival rate

Eleven studies were included (1 mixed and 10 uniform attachments) for this meta-analysis due to the consistency of reported data. The survival analysis for mixed attachments showed an estimated survival rate of 100% (95.3–100%) after 3 years and 5 years (92.3–100%). For uniform attachments, estimated survival rate after 3 years was 99.3% (98.8–99.6%), which was 98.8% after 5 years (98.0–99.4%) (Fig. 2). The difference between mixed and uniform attachment systems was not significant (*p* = 0.302).

### Tooth abutment survival

**Fig. 3:**
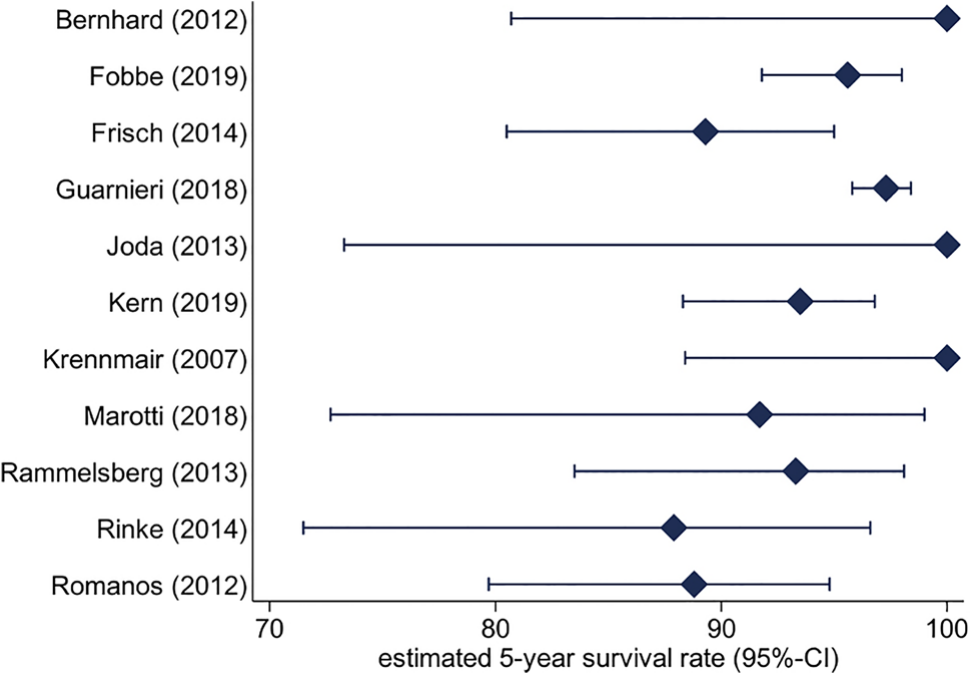
Tooth abutment estimated 5-year survival rate

Tooth abutment survival analysis for mixed attachments was calculated for 11 studies (1 mixed, 10 uniform) and showed an estimated survival rate of 95% (82.6–99.4%) after 3 years and 91.7% after 5 years (72.7–99%). Accordingly, for uniform attachments, the estimated survival rate was 97.2% (96.4–97.8%) after 3 years and 95.4% (94.1–96.4%) after 5 years (Fig. 3). The differences between mixed and uniform attachment systems were not statistically significantly (*p* = 0.45).

### Prosthetic survival rate and complications

**Fig. 4:**
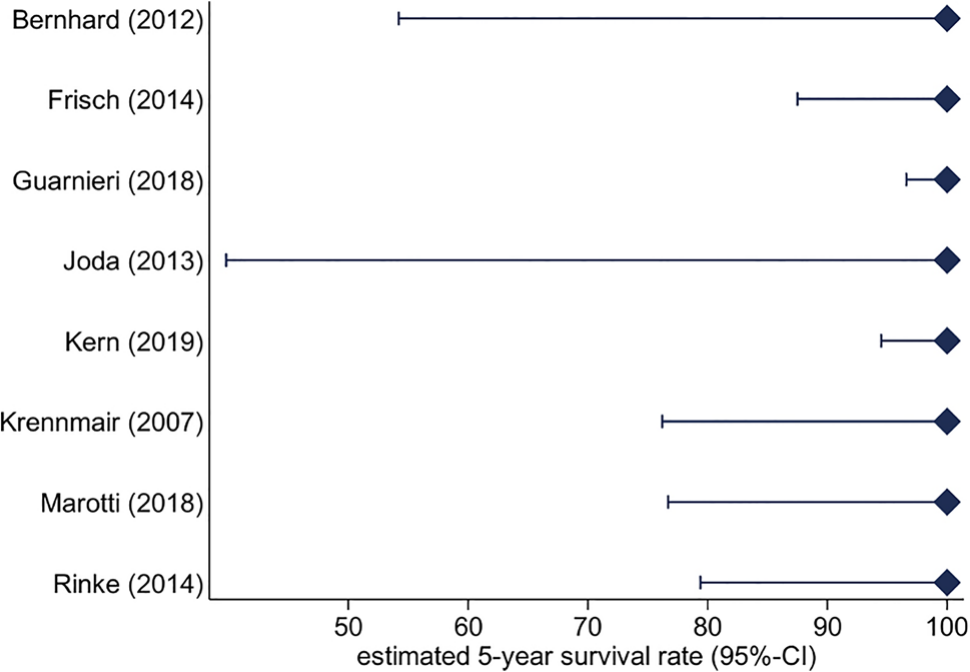
Prosthetic estimated 5-year survival rate

The prosthetic survival rate in 8 studies was calculated to be 100% for mixed and uniform abutments after 3 years and 5 years of function (Fig. 4). Technical complications were reported in 10 studies (1 mixed, 9 uniform), showing a complication rate of 127.3/100 implant years, with statistically significant differences between the treatment modalities in favour of mixed abutment group (*p* < 0.001) (Fig. 4). The mean number of abutments per prosthesis (1.11–4.56 implant abutments; 1.64–6.47 abutment teeth) did not influence the total amount of technical complications (implants IRR = 0.81; *p* = 0.417; teeth IRR = 0.81; *p* = 0.154; implants and teeth IRR = 0.89; *p* = 0.172).

Biological complications for teeth and implants were reported in 10 studies (1 mixed, 9 uniform). Twenty-six biological complications were observed, resulting in a complication rate of 7.7/100 years for the treatment modality with mixed abutments. In uniform abutment group, 243 biological complications were reported and a complication rate of 2/100 years was calculated, which resulted in a statistically significant difference between mixed and uniform attachments (*p* < 0.001). The number of implants per prosthesis (IRR = 0.57; *p* = 0.373), the number of teeth per prosthesis (IRR = 0.86; *p* = 0.272), and the combined number of implants and teeth per prosthesis (IRR = 0.88; *p* = 0.307) did not affect the survival of implant abutments. In contrast, total biological complications were affected by the number of implants per prosthesis (IRR = 0.47; *p* < 0.001), by the number of teeth per prosthesis (IRR = 0.63; *p* < 0.001), and by the combined number of implants and teeth per prosthesis (IRR = 0.74; *p* < 0.001). Studies with more than 5 abutments had a significantly lower rate of biological complications than studies with abutments less than 5 (IRR = 0.57; *p* < 0.001).

For quantitative abutment success, complication evaluation and general and specific information on technical and biological complications and their categorization are summarized in Table 5. Regarding technical complication categories, studies reported 91 minimal, 184 moderate, and 13 severe complications. With regard to biological complications, 68 were minimal, 134 were moderate, and 104 were severe.

**Table 5:** Complications and quality of life reported outcome data of the included studies

Author and year	Technical complication type (*n*)	Technical complications categorized (*n*)	Biological complication type (*n*)	Biological complications categorized (*n*)	QoL measurement tool	Satisfaction value
Bernhart et al. (2012) [21]	1 screw loosening, 2 veneering fracture, and reveneering	3 minimal complications	–	–	NM	NM
Fobbe et al. (2019) [22]	–	–	15 implant loss (peri-implantitis) 19 implants with peri-implantitis 2 apical periodontitis, 3 fracture/endodontic/post-core build-up, 66 acute exacerbation of marginal periodontitis	87 moderate complications, 22 severe complications	NM	NM
Frisch et al. (2015) [23]	7 screw loosening, 2 recementation, 1 loss of retention, 8 veneering fracture, and reveneering or relining	8 minimal and 10 moderate complications	1 implant loss (peri-implantitis), 9 tooth loss (7 caries, 2 endodontic treatment), and 1 crown fracture, 30 peri-implant mucositis	30 minimal and 11 severe complications	NM	NM
Guarnieri and Ippoliti (2018) [24]	6 screw loosening, 3 veneering fracture and reveneering tooth substitution, 34 abutment recementation	6 minimal and 37 moderate complications	6 implant failure, 19 tooth loss (11 periodontal disease, 4 endo-perio lesions, 4 caries)	25 severe complications	NM	NM
Hug et al. (2006) [25]	11 retainer fracture or loss, loss of retention, 2 recementation, 2 female retainer loss, 2 veneering fracture and reveneering, 4 occlusal adjustments	4 minimal and 17 moderate complications	1 root fracture, 2 tooth fracture, 3 sore spots	3 minimal and 3 severe complications	VAS	76–95.5
Joda (2013) [26]	3 screw loosening, 1 veneering fracture, and reveneering	3 minimal and 1 moderate complication	1 endodontic treatment, 2 peri-implant mucosal adverse reaction	3 moderate complications	NM	NM
Kern et al. (2019) [27]	13 screw loosening, 7 relining, 85 veneering fracture and reveneering, 9 recementation, 1 post fracture, 5 fracture of base/framework and renew	20 minimal, 94 moderate, and 6 severe complications	18 caries, 5 pulpitis, 13 peri-implantitis, 10 tooth loss, 2 implant loss, 1 sore spots	1 minimal, 23 moderate, and 12 severe complications	OHIP-14	3–14
Krennmaier et al. (2007) [15]	3 screw loosening, 2 matrix activation, 3 veneering fracture and reveneering, 4 margin adaptation	9 minimal and 3 moderate complications	–	–	NM	NM
Marotti et al. (2015) [28]	20 veneering fracture and reveneering, 7 relining, 7 fracture of base/framework and renew, 25 matrix change, 1 abutment loss and renew	32 minimal, 21 moderate, and 7 severe complications	8 gingivitis, 3 endodontic treatment, 7 caries, 2 tooth extraction, 2 crown fracture, 9 sore spots	17 minimal, 7 moderate, and 4 severe complications	NM	NM
Rammelsberg et al. (2014) [9]	–	–	6 implant loss, 12 mucositis, 11 peri-implantitis, 4 tooth loss, 2 marginal/apical periodontitis, 1 fracture	12 minimal, 13 moderate, 11 severe complications	NM	NM
Rinke et al. (2015) [29]	4 screw loosening, 1 abutment loos, 1 denture repair, 1 retainer fracture or loss	6 minimal and 1 moderate complication	4 tooth fracture, 5 peri-implant mucositis, 1 peri-implantitis	5 Minimal, 1 moderate and 4 severe complications	NM	NM
Romanos et al. (2012) [30]	Relining	Minimal complication	9 tooth extractions, 3 implant failure	12 severe complications	NM	NM

*QoL* quality of life reported outcomes, *NM* not mentioned, *VAS* visual analogic scale, *OHIP-14* Oral Health Impact Profile-14

## Discussion

Considering the obtained results, IARPDs showed excellent survival rates for RPDs, implants, and the implant and abutment teeth with low rates of complications. No influence of combining different types of attachments could be identified. Studies with more than 5 abutments had a significantly lower rate of biological complications than studies that involved abutments less than 5.

Earlier studies have systematically evaluated the use of implants to provide additional support to RPDs [10, 12, 31–34] and prognosis when teeth and implants were combined [10]. However, to the knowledge of the authors, this is the first systematic review that evaluated the clinical performance of the abutments for combined tooth and implant-supported RPDs with different attachment systems. IARPDs could be a viable treatment alternative for partially edentulous patients compared with conventional RPDs. Furthermore, considering the option of extracting remaining teeth for implant overdentures, IARPDs represent an alternative that preserves remaining dentition, enabling the maintenance of alveolar bone and dental proprioception [35, 36].

In terms of implant survival, the results confirm the data from previous studies on IARPDs [2, 10]. Implant-supported FPDs show high survival rates of 95.6% after 5 years and 93.1% after 10 years [37]. Considering similar previous studies and the present data, IARPDs may be especially indicated when fixed reconstructions are not feasible due to financial, anatomical, or other patient-specific factors. Regarding the findings related to the abutment choice, the data for uniform and mixed attachment systems showed no statistical difference, although most of the included studies described the use of telescopic crowns.

The reported data showed that the prognosis of telescopic crown abutments and their dentures is similar to that in previous systematic reviews, reporting favorable results for 3 years and 5 years of follow-ups. Verma et al. [2] assessed the clinical performance of double crown–retained IARPDs which showed tooth survival rates ranging from 82.5 to 96.5% and from 66.7 to 98.6% after 3.4 to 6 years of follow-ups. These results were less promising than those reported in the present study; nevertheless, reported implant survival rates were similar with even a longer follow-up period in Verma et al.’s [2] review. Although the outcomes with telescopic crowns are favorable, high-cost and technique-sensitive procedures are involved, and they are not commonly used worldwide [2, 10].

Biological complications were influenced by the total number of implants and teeth per prosthesis; dentures with 5 or more abutments resulted in improved clinical performance with less biological complications. It was previously reported that the number of abutments for double crowns is critical to ensure an improved prognosis for RPDs [10, 38–40]. Lian et al. [10] reported that these reconstruction prognoses can be improved with more than 3 abutment teeth in combination with implants. When technical and biological complications were individually assessed, the included studies reported a higher rate of biological complications. A potential reason for this outcome may be the inclusion of abutments with questionable prognosis in IARPDs. Such teeth would mostly not be included in fixed restorations as tooth loss may require remake of the restoration, whereas such an issue can be relatively easily resolved when removable prostheses are used. In this regard, this factor should be considered since periodontal, caries, or endodontic status may influence the patient’s oral health compliance [35, 41, 42]. The removable prosthetic component can provide additional hygiene benefits, since patients treated with tooth implant–supported RPDs present a tendency to require few clinical maintenance procedures [10]. Nevertheless, these findings should be interpreted with caution due to risk factors such as previous periodontal disease history that was not mentioned in detail.

IARPDs should have strategic implant distribution to avoid undesirable occlusal forces, and in this regard, opposing arch’s situation may determine the clinical performance of both abutments and prostheses [43]. Five articles reported the use of RPDs, fixed partial dentures, or complete dentures in opposing arch [15, 23, 24, 28–30]. However, due to the heterogeneity of obtained data, the influence of opposing arch could not be analyzed.

It should be mentioned that implant tooth connection for retention could be related with the abutments’ bending capabilities; biomechanical aspects should be considered. Moreover, abutment distribution should be taken into consideration, since increasing the number of abutments can provide a polygonal supplementary prosthetic support scheme [26]. When abutment distribution is considered, a quadrangular arrangement provides more favorable force distribution compared with a linear or triangular design, particularly in the tangential or cross arch linear mandibular or maxillary arches [26, 43]. Although the present study intended to analyze the impact of abutment distribution on clinical outcomes, the included studies were not sufficient to perform such analysis. Hence, further studies focused on the effect of opposing arch, abutment type, and abutment distribution could provide interesting outcomes.

Among the included studies, no randomized clinical trials focusing on the attachment type comparison were available, which is a limitation when analyzing the effect of attachment type. Most of the studies reported the use of telescopic crowns, complicating abutment type comparisons [25, 28]. Nevertheless, all assessed studies were longitudinal with sufficient follow-up to provide 3-year and 5-year assessments. The aforementioned results should be carefully interpreted, since included studies mostly reported on the use of telescopic crowns above all other abutment types. Telescopic crowns seem to be the favorable abutment of choice in IARPDs; however, it should be mentioned that this option is clinically and technically complex and more expensive compared with the use of stock abutments due to individual fabrication of telescopic crowns [2, 10, 38, 40]. Therefore, stock abutments could reduce treatment costs, especially considering the similarity of the results of the present study. Due to the small number of studies that have investigated stock abutments, further studies are needed to support this assumption.

## Conclusions

The use of IARPDs is a viable treatment option for partially edentulous patients, with excellent prosthetic and implant survival rates and favorable rates for abutments after 3-year and 5-year follow-ups. Complications may be reduced when 5 or more abutments are used. Further studies with readily available implant abutments such as ball attachments are needed to corroborate these findings and to reflect clinical outcomes, especially as the number of included studies on mixed attachments was limited.

## Supplementary Information

Below is the link to the electronic supplementary material.

Supplementary file1 (DOCX 24 KB)
