# Supplementary material for: Clinical performance of tooth implant–supported removable partial dentures: a systematic review and meta-analysis
Source: Clin Oral Investig. 2022 Jul 15;26(10):6003–14. doi: 10.1007/s00784-022-04622-7 (PMC9525404; doi:10.1007/s00784-022-04622-7)
Supplement: Supplementary file 1 — Supplementary file1 (DOCX 24 KB) [file 784_2022_4622_MOESM1_ESM.docx]

**Annex 1.** Study excluded with reasons

| **Study** | **Reference number** | **Reason for exclusion** |
| --- | --- | --- |
| Wenz et al. 1998 | 1 | Not enough data |
| Eckert et al. 1998 | 2 | Implant-supported fixed dental prostheses |
| Becker et al. 1999 | 3 | Not enough data |
| Wennenberg et al. 1999 | 4 | Implant-supported fixed dental prostheses |
| Astrand et al. 1999 | 5 | Implant-supported fixed dental prostheses |
| Hallman et al. 2001 | 6 | Implant retained overdenture |
| Aquilino 2001 | 7 | No tooth-implant assisted removable partial denture |
| Hardt et al. 2002 | 8 | Implant-supported fixed dental prostheses |
| Mitrani et al. 2003 | 9 | Not enough data |
| Mijitrinsky et al. 2005 | 10 | Not enough data |
| Mengel et al. (a) 2007 | 11 | Implant-supported fixed dental prostheses |
| Mengel et al. (b) 2007 | 12 | Nor implant or prosthodontic data |
| Alsaadi et al. 2007 | 13 | No tooth-implant assisted removable partial denture |
| Nikening et al 2008 | 14 | Mixed results |
| Grossmann et al. 2008 | 15 | No abutment teeth |
| Kauffman et al. 2009 | 16 | Not exact data |
| Grossman et al. 2009 | 17 | Not exact data |
| Mohammed et al. 2010 | 18 | Not enough data |
| Zafiropoulos et al. 2010 | 19 | 3 patients |
| Katsoulis J et al. 2011 | 20 | Not enough Teeth data |
| Wismeijer et al. 2011 | 21 | Not enough clinical data |
| Bortolini et al. 2011 | 22 | Not enough data |
| Schwarz et al. 2012 | 23 | Not enough data |
| Romanos et al. 2012 | 24 | Edentulous patients |
| Stegelmann et al 2012 | 25 | No implants |
| Wolftart et al. 2012 | 26 | Not enough data |
| Al-Nawas et al. 2012 | 27 | No tooth-implant assisted removable partial denture |
| El Mekawy et al. 2012 | 28 | Not enough data |
| Mangano et al. 2014 | 29 | No tooth-implant assisted removable partial denture |
| Mayle et al. 2014 | 30 | Implant-supported fixed dental prostheses |
| Heitor Campos et al. 2015 | 31 | Not enough follow-up |
| Mostafa et al. 2015 | 32 | Implant-supported fixed dental prostheses |
| Jensen et al. 2016 | 33 | No abutment teeth |
| Mundt et al. 2016 | 34 | No results |
| Wolfart et al. 2016 | 35 | No follow-up mentioned |
| Pozzi et al. 2016 | 36 | Implant-supported fixed dental prostheses |
| Tabrizi et al. 2017 | 37 | Implant-supported fixed dental prostheses |
| Giacomel et al. 2017 | 38 | Implant-supported fixed dental prostheses |
| Karlsson et al. 2018 | 39 | No detailed data |
| Jemt T. 2019 | 40 | Implant-supported fixed dental prostheses |
| Wigl et al. 2019 | 41 | Edentulous patients |
| Guaerneri et al. 2019 | 42 | Edentulous patients |
| Adler et al. 2019 | 43 | Implant-supported fixed dental prostheses |
| Bäumer et al. 2020 | 44 | Implant-supported fixed dental prostheses |

1. Wenz HJ, Lehmann KM. A telescopic crown concept for the restoration of the partially edentulous arch: the Marburg double crown system. Int J Prosthodont. 1998 Nov-Dec;11(6):541-50. PMID: 10023216.
2. Eckert SE, Wollan PC. Retrospective review of 1170 endosseous implants placed in partially edentulous jaws. J Prosthet Dent. 1998 Apr;79(4):415-21. doi: 10.1016/s0022-3913(98)70155-6.
3. Becker W, Becker BE, Alsuwyed A, Al-Mubarak S. Long-term evaluation of 282 implants in maxillary and mandibular molar positions: a prospective study. J Periodontol. 1999 Aug;70(8):896-901. doi: 10.1902/jop.1999.70.8.896.
4. Wennerberg A, Jemt T. Complications in partially edentulous implant patients: a 5-year retrospective follow-up study of 133 patients supplied with unilateral maxillary prostheses. Clin Implant Dent Relat Res. 1999;1(1):49-56. doi: 10.1111/j.1708-8208.1999.tb00091.x. PMID: 11359311.
5. Astrand P, Engquist B, Dahlgren S, Gröndahl K, Engquist E, Feldmann H. Astra Tech and Brånemark system implants: a 5-year prospective study of marginal bone reactions. Clin Oral Implants Res. 2004 Aug;15(4):413-20. doi: 10.1111/j.1600-0501.2004.01028.x.
6. Hallman M. A prospective study of treatment of severely resorbed maxillae with narrow nonsubmerged implants: results after 1 year of loading. Int J Oral Maxillofac Implants. 2001 Sep-Oct;16(5):731-6.
7. Aquilino SA, Shugars DA, Bader JD, White BA. Ten-year survival rates of teeth adjacent to treated and untreated posterior bounded edentulous spaces. J Prosthet Dent. 2001 May;85(5):455-60. doi: 10.1067/mpr.2001.115248.
8. Hardt CR, Gröndahl K, Lekholm U, Wennström JL. Outcome of implant therapy in relation to experienced loss of periodontal bone support: a retrospective 5- year study. Clin Oral Implants Res. 2002 Oct;13(5):488-94. doi: 10.1034/j.1600-0501.2002.130507.x.
9. Mitrani R, Brudvik JS, Phillips KM. Posterior implants for distal extension removable prostheses: a retrospective study. Int J Periodontics Restorative Dent. 2003 Aug;23(4):353-9.
10. Mijiritsky E, Ormianer Z, Klinger A, Mardinger O. Use of dental implants to improve unfavorable removable partial denture design. Compend Contin Educ Dent. 2005 Oct;26(10):744-6, 748, 750 passim. PMID: 16231543.
11. Mengel R, Behle M, Flores-de-Jacoby L. Osseointegrated implants in subjects treated for generalized aggressive periodontitis: 10-year results of a prospective, long-term cohort study. J Periodontol. 2007 Dec;78(12):2229-37. doi: 10.1902/jop.2007.070201.
12. Mengel R, Lehmann KM, Metke W, Wolf J, Flores-de-Jacoby L. A telescopic crown concept for the restoration of partially edentulous patients with aggressive generalized periodontitis: two case reports. Int J Periodontics Restorative Dent. 2002 Apr;22(2):129-37. PMID: 12019708.
13. Alsaadi G, Quirynen M, Komárek A, van Steenberghe D. Impact of local and systemic factors on the incidence of oral implant failures, up to abutment connection. J Clin Periodontol. 2007 Jul;34(7):610-7. doi: 10.1111/j.1600-051X.2007.01077.x.
14. Nickenig HJ, Spiekermann H, Wichmann M, Andreas SK, Eitner S. Survival and complication rates of combined tooth-implant-supported fixed and removable partial dentures. Int J Prosthodont. 2008 Mar-Apr;21(2):131-7.
15. Grossmann Y, Levin L, Sadan A. A retrospective case series of implants used to restore partially edentulous patients with implant-supported removable partial dentures: 31-month mean follow-up results. Quintessence Int. 2008 Sep;39(8):665-71. PMID: 19107253.
16. Kaufmann R, Friedli M, Hug S, Mericske-Stern R. Removable dentures with implant support in strategic positions followed for up to 8 years. Int J Prosthodont. 2009 May-Jun;22(3):233-41; discussion 242. PMID: 19548404.
17. Grossmann Y, Nissan J, Levin L. Clinical effectiveness of implant-supported removable partial dentures: a review of the literature and retrospective case evaluation. J Oral Maxillofac Surg. 2009 Sep;67(9):1941-6. doi: 10.1016/j.joms.2009.04.081.
18. Mohamed GF, El Sawy AA. The role of single immediate loading implant in long Class IV Kennedy mandibular partial denture. Clin Implant Dent Relat Res. 2012 Oct;14(5):708-15. doi: 10.1111/j.1708-8208.2010.00305.x.
19. Zafiropoulos GG, Rebbe J, Thielen U, Deli G, Beaumont C, Hoffmann O. Zirconia removable telescopic dentures retained on teeth or implants for maxilla rehabilitation. Three-year observation of three cases. J Oral Implantol. 2010;36(6):455-65. doi: 10.1563/AAID-JOI-D-09-00065.
20. Katsoulis J, Nikitovic SG, Spreng S, Neuhaus K, Mericske-Stern R. Prosthetic rehabilitation and treatment outcome of partially edentulous patients with severe tooth wear: 3-years results. J Dent. 2011 Oct;39(10):662-71. doi: 10.1016/j.jdent.2011.07.008
21. Wismeijer D, Tawse-Smith A, Payne AG. Multicentre prospective evaluation of implant-assisted mandibular bilateral distal extension removable partial dentures: patient satisfaction. Clin Oral Implants Res. 2013 Jan;24(1):20-7. doi: 10.1111/j.1600-0501.2011.02367.x.
22. Bortolini S, Natali A, Franchi M, Coggiola A, Consolo U. Implant-retained removable partial dentures: an 8-year retrospective study. J Prosthodont. 2011 Apr;20(3):168-72. doi: 10.1111/j.1532-849X.2011.00700.x.
23. Schwarz S, Bernhart G, Hassel AJ, Rammelsberg P. Survival of double-crown-retained dentures either tooth-implant or solely implant-supported: an 8-year retrospective study. Clin Implant Dent Relat Res. 2014 Aug;16(4):618-25. doi: 10.1111/cid.12023.
24. Romanos GE, May S, May D. Implant-supporting telescopic maxillary prostheses and immediate loading. Clin Implant Dent Relat Res. 2014 Jun;16(3):412-8. doi: 10.1111/cid.12003.
25. Stegelmann K, Dirheimer M, Ludwig E, Moldovan O, Rudolph H, Luthardt RG, Just BA. Case-control study on the survival of abutment teeth of partially dentate patients. Clin Oral Investig. 2012 Dec;16(6):1685-91. doi: 10.1007/s00784-011-0661-5.
26. Wolfart S, Moll D, Hilgers RD, Wolfart M, Kern M. Implant placement under existing removable dental prostheses and its effect on oral health-related quality of life. Clin Oral Implants Res. 2013 Dec;24(12):1354-9. doi: 10.1111/clr.12030.
27. Al-Nawas B, Kämmerer PW, Morbach T, Ladwein C, Wegener J, Wagner W. Ten-year retrospective follow-up study of the TiOblast dental implant. Clin Implant Dent Relat Res. 2012 Mar;14(1):127-34. doi: 10.1111/j.1708-8208.2009.00237.x.
28. El Mekawy NH, El-Negoly SA, Grawish Mel-A, El-Hawary YM. Intracoronal mandibular Kennedy Class I implant-tooth supported removable partial overdenture: a 2-year multicenter prospective study. Int J Oral Maxillofac Implants. 2012 May-Jun;27(3):677-83. PMID: 22616063.
29. Mangano F, Shibli JA, Sammons RL, Veronesi G, Piattelli A, Mangano C. Clinical outcome of narrow-diameter (3.3-mm) locking-taper implants: a prospective study with 1 to 10 years of follow-up. Int J Oral Maxillofac Implants. 2014 Mar-Apr;29(2):448-55. doi: 10.11607/jomi.3327.
30. Meyle J, Gersok G, Boedeker RH, Gonzales JR. Long-term analysis of osseointegrated implants in non-smoker patients with a previous history of periodontitis. J Clin Periodontol. 2014 May;41(5):504-12. doi: 10.1111/jcpe.12237.
31. Campos CH, Gonçalves TM, Garcia RC. Implant-Supported Removable Partial Denture Improves the Quality of Life of Patients with Extreme Tooth Loss. Braz Dent J. 2015 Oct;26(5):463-7. doi: 10.1590/0103-6440201300097.
32. Mostafa TM, El-Sheikh MM, Abd El-Fattah F. Implant-connected versus tooth-connected implant-supported partial dentures: 2-year clinical and radiographic comparative evaluation. Int J Periodontics Restorative Dent. 2015 May-Jun;35(3):335-43. doi: 10.11607/prd.2310.
33. Jensen C, Meijer HJA, Raghoebar GM, Kerdijk W, Cune MS. Implant-supported removable partial dentures in the mandible: A 3-16 year retrospective study. J Prosthodont Res. 2017 Apr;61(2):98-105. doi: 10.1016/j.jpor.2016.07.002.
34. Mundt T, Al Jaghsi A, Schwahn B, et al. Immediate versus delayed loading of strategic mini dental implants for the stabilization of partial removable dental prostheses: a patient cluster randomized, parallel-group 3-year trial. BMC Oral Health. 2016;17(1):30. Published 2016 Jul 30. doi:10.1186/s12903-016-0259-z
35. Wolfart S, Wolf K, Brunzel S, Wolfart M, Caliebe A, Kern M. Implant placement under existing removable dental prostheses and its effect on masticatory performance. Clin Oral Investig. 2016 Dec;20(9):2447-2455. doi: 10.1007/s00784-016-1746-y.
36. Pozzi A, Mura P. Immediate Loading of Conical Connection Implants: Up-to-2-Year Retrospective Clinical and Radiologic Study. Int J Oral Maxillofac Implants. 2016 Jan-Feb;31(1):142-52. doi: 10.11607/jomi.4061.
37. Tabrizi R, Behnia H, Taherian S, Hesami N. What Are the Incidence and Factors Associated With Implant Fracture? J Oral Maxillofac Surg. 2017 Sep;75(9):1866-1872. doi: 10.1016/j.joms.2017.05.014.
38. Giacomel MC, Camati P, Souza J, Deliberador T. Comparison of Marginal Bone Level Changes of Immediately Loaded Implants, Delayed Loaded Nonsubmerged Implants, and Delayed Loaded Submerged Implants: A Randomized Clinical Trial. Int J Oral Maxillofac Implants. 2017 May/Jun;32(3):661-666. doi: 10.11607/jomi.5353.
39. Karlsson K, Derks J, Håkansson J, Wennström JL, Molin Thorén M, Petzold M, Berglundh T. Technical complications following implant-supported restorative therapy performed in Sweden. Clin Oral Implants Res. 2018 Jun;29(6):603-611. doi: 10.1111/clr.13271.
40. Jemt T. Implant Survival in the Posterior Partially Edentulous Arch-30 Years of Experience. Part IV: A Retro-Prospective Multivariable Regression Analysis on Implant Failures Related to Arch and Implant Surface. Int J Prosthodont. 2019 Mar/Apr;32(2):143-152. doi: 10.11607/ijp.6012
41. Weigl P, Trimpou G, Lorenz J, Nentwig GH, Lauer HC. Prefabricated taper crowns for the retention of implant superstructures: Three-year results of a prospective clinical trial. J Prosthet Dent. 2019 Apr;121(4):618-622. doi: 10.1016/j.prosdent.2018.07.004.
42. Guarnieri R, Di Nardo D, Di Giorgio G, Miccoli G, Testarelli L. Full arch fixed prostheses vs. full arch telescopic-retained retrievable prostheses both supported by implants and natural tooth abutments in periodontally treated patients: Results at 15 years. J Clin Exp Dent. 2019 Oct 1;11(10):e937-e946. doi: 10.4317/jced.55904.
43. Adler L, Buhlin K, Jansson L. Survival and complications: A 9- to 15-year retrospective follow-up of dental implant therapy. J Oral Rehabil. 2020 Jan;47(1):67-77. doi: 10.1111/joor.12866.
44. Bäumer A, Toekan S, Saure D, Körner G. Survival and success of implants in a private periodontal practice: a 10 year retrospective study. BMC Oral Health. 2020 Mar 30;20(1):92. doi: 10.1186/s12903-020-01064-z.
